# Depressive symptoms among Olympic athletes during the Covid-19 pandemic

**DOI:** 10.1186/s13102-022-00427-z

**Published:** 2022-03-10

**Authors:** Christophe Lambert, Lisa-Marie Schuetz, Simon Rice, Rosemary Purcell, Theresa Stoll, Martyna Trajdos, Ramona Ritzmann, Anna-Lena Böhm, Martin Walz

**Affiliations:** 1 Department of Trauma and Orthopedic Surgery, Cologne Merheim Medical Center, University of Witten/Herdecke, Classen-Kappelmann Str. 25, 50931 Cologne, Germany; 2 Institute of Sports and Sports Sciences, Heidelberg University, Heidelberg, Germany; 3 Orygen, Parkville, Australia; 4 Centre for Youth Mental Health, The University of Melbourne, Melbourne, VIC Australia; 5 TUM School of Medicine, Technical University of Munich, Munich, Germany; 6 Institute of Movement Therapy and Movement-Oriented Prevention and Rehabilitation, German Sport University Cologne, Cologne, Germany; 7 Department of Sports and Sports Sciences, University of Freiburg, Freiburg, Germany; 8 Faculty of Psychology, Distance University of Hagen, Hagen, Germany; 9 Chair of Sport Psychology, TUM Department of Sport and Health Sciences, Technical University of Munich, Munich, Germany

**Keywords:** COVID-19 pandemic, Olympic Games Tokyo, Mental health, Athletes

## Abstract

**Objectives:**

The aim of this study was to analyze athlete-specific psychological strain among Olympic athletes following the postponement of the Tokyo 2020 Olympic games due to the COVID-19 pandemic.

**Methods:**

A survey that comprised three sub-sections (Psychological Strain Questionnaire (APSQ), Patient Health Questionnaire—Depression Module (PHQ-8) and Participant characteristic) concerning mental health, performance issues and concerns about the postponement of the Tokyo Olympics, was distributed online and sent to 102 Olympic athletes.

**Results:**

A total of 85 participants from 11 Olympic sports were enrolled. Results indicated that most athletes showed psychological strain related to concerns regarding the postponement of the Tokyo Olympics. Depression severity was positively associated with maladaptive avoidance coping patterns, negative effects in training, worries and fear. Depression severity was also negatively associated with motivation and adaptive factors such as chances and opportunities that can be drawn from the pandemic.

**Conclusion:**

The present sample of Olympic athletes reported suffering from psychological uncertainty associated with the postponement of the Olympic games. Sports federations should therefore, provide ongoing wellbeing support to athletes and offer them, for example, sports psychological support in order to be able to better deal with pandemic-related uncertainties and changes.

## Introduction

The International Society of Sport Psychology suggested that the postponement of the 2020 Tokyo Olympic games represents a significant career disruption that could involve a loss of identity, motivation and meaning [1] with emotional responses that may stimulate a series of mental health issues according to whether athletes are late or early in their careers.

The emotional response of Olympic and Paralympic athletes to the decision to postpone the Olympic games in Tokyo is likely to be varied [2]. After years of preparing for the event, the prospect of postponement, let alone potential cancelation, can lead to negative emotional responses, such as prolonged physical and psychological pressure, lack of motivation, concerns about future performance, disappointment, instability and confusion to one’s athletic identity [3]. Conversely, for some athletes, postponement of the games was associated with relief, mental release, an opportunity for better preparation and personal enrichment [4, 5].

While the severe acute respiratory syndrome coronavirus 2 (hereafter referred as COVID-19) continues to spread worldwide, the impact on mental health symptoms and disorders in both the general population and within elite sports is gaining increased attention [6–8]. Xiong et al. [9] revealed with their systematic review an increase of physiological distress in the general population since the COVID-19 pandemic [9]. Elite athletes have been shown in pre-pandemic studies to experience mental health symptoms and disorders at a level equivalent to, or in some cases exceeding the non-athlete population [10, 11]. The consensus statement of the International Olympic Committee on mental health symptoms described specific mental health symptoms and disorders in eltite athletes as sleep disorders and sleep concerns, major depressive disorder and depression symptoms, suicide, anxiety and related disorders, post-traumatic stress disorder and other trauma-related disorders, eating disorders, attention-deficit/hyperactivity disorder, bipolar and psychotic disorders, sport-related concussion, substance use and substance use disorders, gambling disorder and other behavioral addictions [10]. Every of these mentioned conditions have been proven in the literature to occur in elite athletes. It seems that a high number of athletes are avoiding to talk about these topics and that in elite sports it is not common to ask for help. The picture that an athlete has to be strong and show no weakness is still very present [12]. It could be reasonable to assume that the COVID-19 pandemic and the consequences for training and competition may create an additional burden on the mental health and well-being of Olympic athletes.

This study aimed to explore and describe mental health symptoms and athlete-specific psychological strain among Olympic athletes during this pandemic phase, and to understand whether sports psychology, medical and/or coaching support were available for athletes during this phase. We hypothesized that the postponement of the Olympic Games would have a negative impact on athlete wellbeing, as evidenced by elevated levels of psychological strain and depressive symptoms.

## Methods

### Participants and procedures

The present study was part of a research project examining dispositional and emotional features associated with athletes’ mental health symptoms due to the COVID-19 pandemic. An online questionnaire was distributed by international sports federations with the request to send it to athletes who had already qualified or were still in the qualification process for the Olympic Games in Tokyo 2020. The survey was send to 102 athletes from which 86 (84%) completed the survey. One participant did not agree to the privacy policy statement and was excluded.

The online survey was open from July until September 2020.

Inclusion criteria:

- Athletes who had already qualified or were still in the qualification process for the Olympic Games in Tokyo 2020
- Age 18 years and older
- Complete survey
- Agreement to the privacy policy statement

Exclusion criteria:

- Athletes who had not qualified or were not involved in the qualification process for the Olympic Games in Tokyo 2020
- Age under 18 years
- Incomplete survey
- No agreement to the privacy policy statement

Participants provided informed consent before participating voluntarily in the study. The study was approved by the Ethics Committee of the University of Witten/Herdecke (No. 171/2020). The study team used the Declaration of Helsinki ethical principles for medical research involving human subjects as standards.

### Instruments

The Survey comprised three sub-sections, including participant demographics, questions related to the COVID-19 pandemic and 41 questions about athletes’ feelings towards their sporting activity.

#### Athlete Psychological Strain Questionnaire (APSQ)

The APSQ is a 10-item mental health questionnaire with 3 subscales assessing self-regulation, performance and external coping in athletes. The 4-item performance subscale and 3 additional single items from APSQ (two items from subscale self-regulation: “Did you feel less motivated?”, “Did you feel irritable, angry or aggressive?”; and one item concerning external coping: “Did you need alcohol or other substances to relax?”) were used to provide a brief, targeted self-report of athletic distress and maladaptive avoidance coping patterns (such as the use of alcohol) [13]. Items were reworded to specifically address the athletes stress and coping arising from the COVID-19 pandemic (e.g. “Did you find training more stressful?”, “Did you think you could cope with the difficult COVID-19 situation?”). Given the context for the study, 3 items were excluded from the questionnaire (“It was difficult to be around teammates”, “I found it difficult to do what I needed to do” and “I took unusual risks off-field”). As the psychometrically validated 10-item APSQ was not utilized (as not all subscales were used), the APSQ items were instead analyzed as single items, using the existing 5-point Likert response scale (1 = none of the time, to 5 = all of the time).

#### Patient Health Questionnaire—Depression Module (PHQ-8)

The PHQ-8 was utilized as a valid diagnostic and severity measure for depressive disorders [14]. It consists of eight of the nine criteria (omitting suicide and self-harm risk) on which the DSM-IV diagnosis of depressive disorders is based. Using a 4-point Likert scale, athletes rated the severity of each depressive symptom from “not at all” to “nearly every day”. 13% (*n* = 11) of the athletes scored the commonly used threshold of ≥ 10 for the PHQ-8. Scores above this cutpoint suggest that the probability for a major depression is increased [14].

#### Participant characteristic

The third section of the questionnaire included general questions about participants` characteristics, including gender, age, nationality and type of sport. In addition, we asked for travel time for sports in days both before and after the COVID-19 pandemic, along with living conditions and the injury status (e.g. “Do you live alone or with a partner, family or friends?”, “Are you currently injured?”). Participants were also asked questions about the postponement of the Olympics and resulting consequences (e.g. “Was it difficult for you when the Tokyo Olympics 2020 were postponed?”, “Did you see this time as a chance to be even better prepared for the Olympics 2021?”, “Do you think it was the right decision to postpone the Tokyo Olympics 2020?”, “Did you have psychological help to cope with the situation?”). Participants answered using a 5-point Likert scale from 1 = “None of the time” to 5 = “all of the time”.

#### Data availability statement

The data that support the findings of this study are available from the corresponding author, [CL], upon reasonable request.

### Statistical analysis

To investigate associations between depression and athletes’ problems, worries and fears, Pearson correlation analyses were conducted. We conducted *t*-tests to explore mean differences between athletes. For these *t*-tests the five response categories "None of the time", "A little of the time", "Some of the time", "Most of the time" and "All of the time" of single items were combined into dummy variables with the category "rarely" for the first two response categories and with the category "yes" for the last three response categories. Whenever group size (in the *t*-test) was smaller than 30 nonparametric tests were calculated additionally. Results did not show any differences regarding significance. Effect sizes according to Hedge's *g* were determined for all differences. All statistical analyses were performed using IBM SPSS 27 (Chicago, IL, USA), with *p*-values < 0.05 used as significance threshold. All plots were created using the R package [ggplot2, scales].

## Results

### Study population

**Fig. 1:**
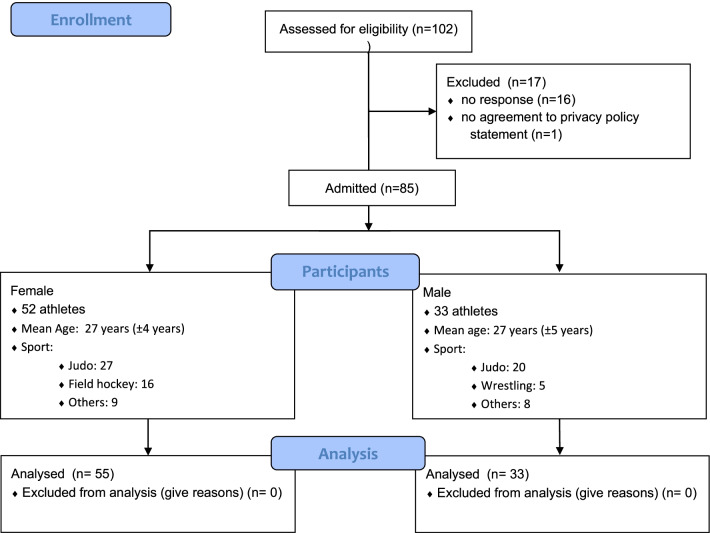
Flow chart diagram of the included athletes separated by gender

A total of 85 participants (52 women), aged 18 to 44 years (*M* = 26.92, *SD* = 4.60) participated. Athletes of 11 Olympic sports and 24 countries took part in the study. Most participants were Olympic athletes in judo (55%; *n* = 47), field hockey (20%; *n* = 17), wrestling (8%; *n* = 7) and surfing (7%; *n* = 6). For most, (60%, *n* = 51) the Olympics 2020 in Tokyo would have represented their first Olympic games. A total of 40% (n = 34) athletes had participated in a previous Olympics, with 13% (*n* = 11) athletes participating twice or three times. Athletes detailed demographics are shown in the Flow chart diagram in Fig. 1.

### Mental symptoms correlated towards the postponement of the Olympic games

Almost all participants (93%; *n* = 79) reported agreeing with the decision to postpone the Tokyo Olympic Games (*M* = 4.48, *SD* = 0.91; higher numbers indicate higher consent). While a majority of athletes (65%; *n* = 55) reported experiencing difficulties when the Olympic games were postponed (*M* = 2.86, *SD* = 1.10), most (82%; *n* = 70) also reported seeing an opportunity to better prepare for the re-scheduled Olympics in 2021 (*M* = 3.56, *SD* = 1.13) and to work on their weakness (77%; *n* = 65; *M* = 3.44, *SD* = 1.19). Means and standard deviations from single items concerning feelings about the postponement, training and coping can be found in Table1.

**Table 1:** Means and standard deviations from single items concerning feelings about the postponement, training and coping due the postponement of the Olympic Games

Variables		
Was it difficult for you when the Tokyo Olympics 2020 were postponed?	*M* (*SD*)	2.86 (1.10)
Did you see this time as a chance to be even better prepared for the Olympics 2021?	*M* (*SD*)	3.56 (1.13)
Did you see this time without competitions as a chance to work on your weaknesses?	*M* (*SD*)	3.44 (1.19)
Did the COVID-19 crisis affect your training?	*M* (*SD*)	3.96 (0.92)
Did you feel less motivated?	*M* (*SD*)	2.79 (1.04)
Did you feel irritable, angry or aggressive?	*M* (*SD*)	2.21 (1.03)
Have you been afraid that athletes from other countries could train better during this time?	*M* (*SD*)	2.85 (1.13)
Have you been afraid that due to your different training you will not be at the same level like your opponents?	*M* (*SD*)	2.69 (1.13)
Did you feel pressure from yourself to get back to “Full-Contact-Training”?	*M* (*SD*)	3.01 (1.13)
Have you been afraid that you could lose your income due to the COVID-19 Crisis?	*M* (*SD*)	2.39 (1.28)
Did you use the free time out of sport to work on your life after your career as an athlete? (Studies, Education)	*M* (*SD*)	3.27 (1.13)
Did you need alcohol or other substances to relax?	*M* (*SD*)	1.21 (0.53)
Did you have psychological help to cope with the situation?	*M* (*SD*)	1.72 (1.09)
Do you think that handling this COVID-19 situation made you mentally stronger	*M* (*SD*)	3.27 (1.00)
Do you think it was the right decision to postpone the Tokyo Olympics 2020?	*M* (*SD*)	4.48 (0.91)
Are you now impatient to start competitions and training camps again?	*M* (*SD*)	3.27 (1.23)

*Note* Answers were made using the 5-point Likert scale from 1 = “None of the time” to 5 = “all of the time” (higher numbers indicate higher consent)

### PHQ scores

Across the sample, the mean PHQ-8 score was 5.51 (*SD* = 3.70). The commonly used cut point of ≥ 10 [14] revealed, that 11 athletes scored above the threshold (*M* = 12.00, *SD* = 2.97, 12.9%) and 74 athletes scored below (*M* = 4.54, *SD* = 2.68, 86.1%). The standard PHQ-8 severity intervals (0–4 no depression, 5–9 mild depression, 10–14 moderate depression, 15–19 moderately severe depression and 20–24 severe depression) indicated that 37 athletes reported no depression (*M* = 2.22, *SD* = 1.38), 37 athletes reported mild depression (*M* = 6.86, *SD* = 1.25), 9 athletes moderate depression (*M* = 10.89, *SD* = 1.54) and 2 athletes a moderately severe depression (*M* = 17.00, *SD* = 2.83). The majority of athletes reported that they had never seen any professional for psychological support (60%, *n* = 51). We did not find any sex differenced when looking at depressiveness scores. When comparing team sport (*n* = 17; *M* = 7.88, *SD* = 4.36) and individual sport (*n* = 68; *M* = 4.91, *SD* = 3.28), we found significant differences in PHQ-8 scores, *t*(83) = 3.12, *p* = 0.003, *g* = 0.84, 95% CI [0.29, 1.38], showing that athletes in team sports report higher PHQ-8 scores than athletes from individual sports.

### APSQ

**Fig. 2:**
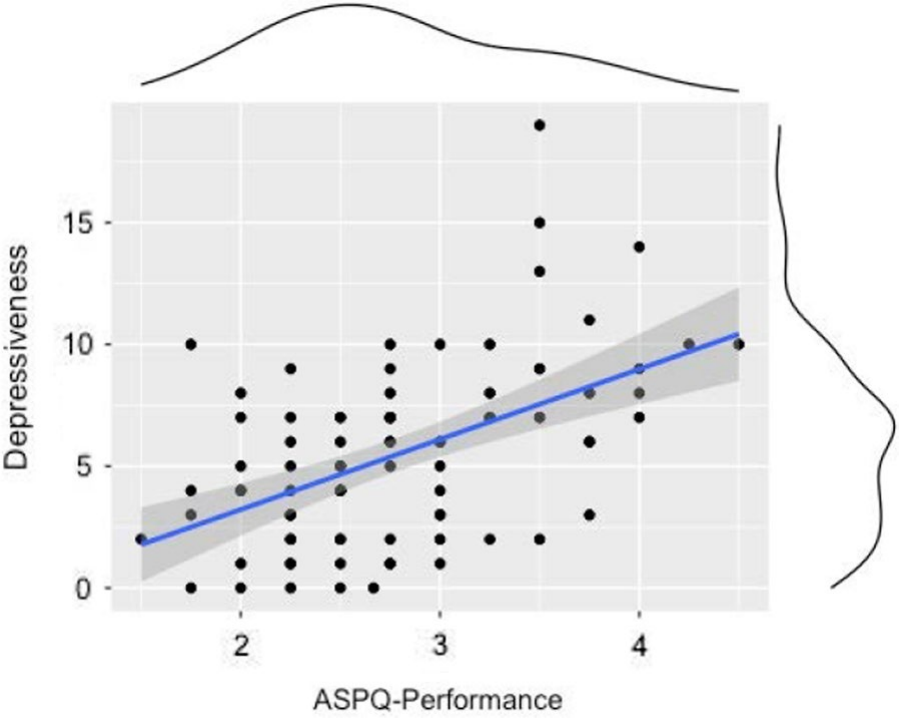
Distribution and the scatterplot of the correlation between APSQ performance subscale and PHQ-8 score

The mean score reported on the performance subscale of the APSQ was 11.14 (*SD* = 2.65: possible range = 4–20). No sex differences were found. PHQ-8 scores correlated strongly with the APSQ performance subscale, *r*(85) = 0.53, *p* = 0.001. Figure 2 shows the items distribution and the scatterplot of the correlation between APSQ performance subscale and PHQ-8 score.

When comparing team sport (*n* = 17; *M* = 3.24, *SD* = 0.82) and individual sport (*n* = 68; *M* = 2.68, *SD* = 0.56), we found significant differences in on the performance subscale of the APSQ, *t*(83) = 3.28, *p* = 0.002, *g* = 0.88, 95% CI [0.33, 1.42], showing that athletes in team sports report higher psychological strain than athletes from individual sports.

### Correlations

In order to determine whether there was an association between depression severity as measured by the PHQ-8 and increased problems during training, self-regulation and athlete’s worries, correlation analyses were conducted. The results show that depression severity was positively associated with more negative effects in training, more worries and fear, less motivation and fewer positive factors such as opportunities that could be drawn from the pandemic (Table 2).

**Table 2:** Correlation between depression severity as measured by the PHQ-8 and increased problems during training, self-regulation and athlete’s worries

	1	2	3	4	5	6	7	8	9	10	11	12	13
1. PHQ-8		0.53**	0.45**	0.33**	0.54**	0.56**	0.44**	0.45**	0.64**	0.32**	0.38**	− 00.20	− 0.31**
2. ASPQ—performance			0.45**	0.44**	0.57**	0.59**	0.55**	0.37**	0.66**	0.35**	0.39**	− 0.25*	− 0.34**
3. Difficulties with the postponement				0.34**	0.29**	0.39**	0.50**	0.24*	0.39**	0.23*	0.36**	− 0.54**	− 0.35**
4. COVID-19 affected training					0.44**	0.37**	0.35**	0.32**	0.26*	0.14	0.22*	− 0.26*	− 0.41**
5. Afraid that others train better						0.74**	0.54**	0.45**	0.46**	0.34**	0.31**	− 00.04	− 0.26*
6. Afraid not to be at the same level							0.49**	0.43**	0.51**	0.40**	0.44**	− 00.19	− 0.34**
7. Self-pressure to get back to								0.41**	0.51**	0.31**	00.21	− 00.14	− 00.21
8. Did you feel less motivated									0.47**	00.21	00.19	− 0.22*	− 0.39**
9. Feel irritable, angry or aggressive										0.30**	0.41**	− 00.20	− 0.26*
10. Afraid of the first competition											− 00.01	− 00.06	− 00.19
11. Afraid to lose income												− 00.13	− 00.08
12. Chance to better prepare													0.57**
13. Chance to work on weaknesses													

*Note* *p < 0.05, **p < 0.01

Athletes who indicated difficulties with the postponement of the Olympics in Tokyo (*n* = 55) reported significantly higher depression scores (*M* = 6.36, *SD* = 3.79) compared to athletes having no difficulties (*n* = 30; *M* = 3.93, *SD* = 2.96), *t*(83) = − 3.04, *p* = 0.003, *g* = − 0.68, 95% CI [− 1.13, − 0.23]. Similar results were obtained in relation to athletes finding training more stressful (*n* = 34; *M* = 6.71, *SD* = 3.88) compared to not (*n* = 51; *M* = 4.71, *SD* = 3.37), *t*(83) = − 2.52, *p* = 0.01, *g* = − 0.55, 95% CI [− 0.99, − 0.11], athletes feeling less motivated (*n* = 49; *M* = 6.35, *SD* = 3.61) compared to those not feeling less motivated (*n* = 36; *M* = 4.36, *SD* = 3.53), *t*(83) = − 2.53, *p* = 0.01, *g* = − 0.55, 95% CI [− 0.98, − 0.11], and athletes feeling irritable, angry or aggressive (*n* = 31; *M* = 8.03, *SD* = 3.88) compared to those feeling rarely irritable, angry or aggressive (*n* = 54; *M* = 4.06, *SD* = 2.68), *t*(83) = − 5.57, *p* < 0.001, *g* = − 1.24, 95% CI [− 1.72, − 0.76]. Athletes seeing this time as an opportunity to work on their weaknesses reported significantly lower depression scores (*n* = 65; *M* = 4.85, *SD* = 3.24) compared to athletes not seeing this chance (*n* = 20; *M* = 7.65, *SD* = 4.31), *t*(83) = 3.12, *p* = 0.002, *g* = 0.79, 95% CI [0.28, 1.30].

**Fig. 3:**
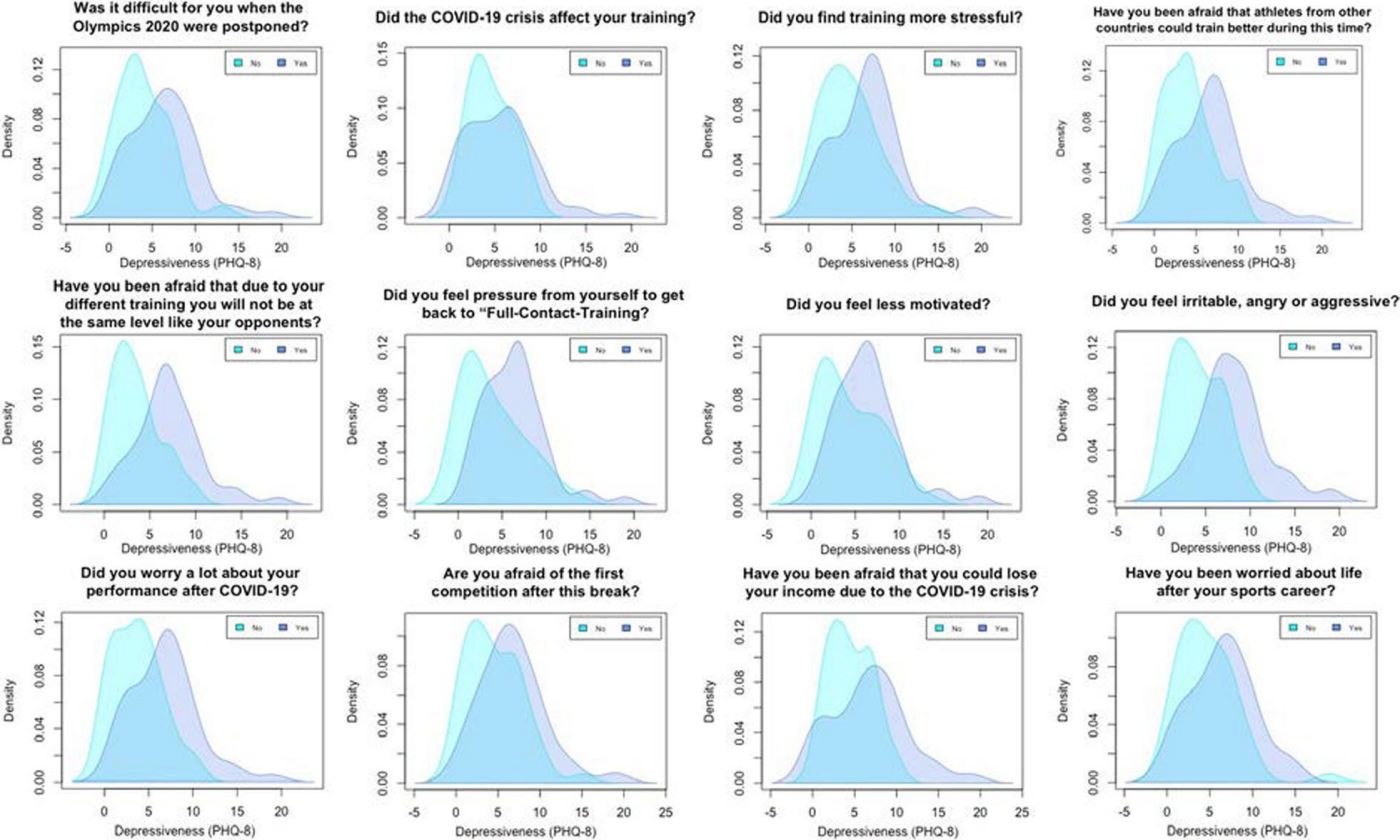
Density distribution

Figure 3 shows the results of the *t*-test analysis. The density distributions suggest that the effect described above is caused by the fact that the PHQ8 score distribution of people answering with “yes” (dummy coded variables) shifted to the right, which suggests higher scores on the PHQ-8.

**Fig. 4:**
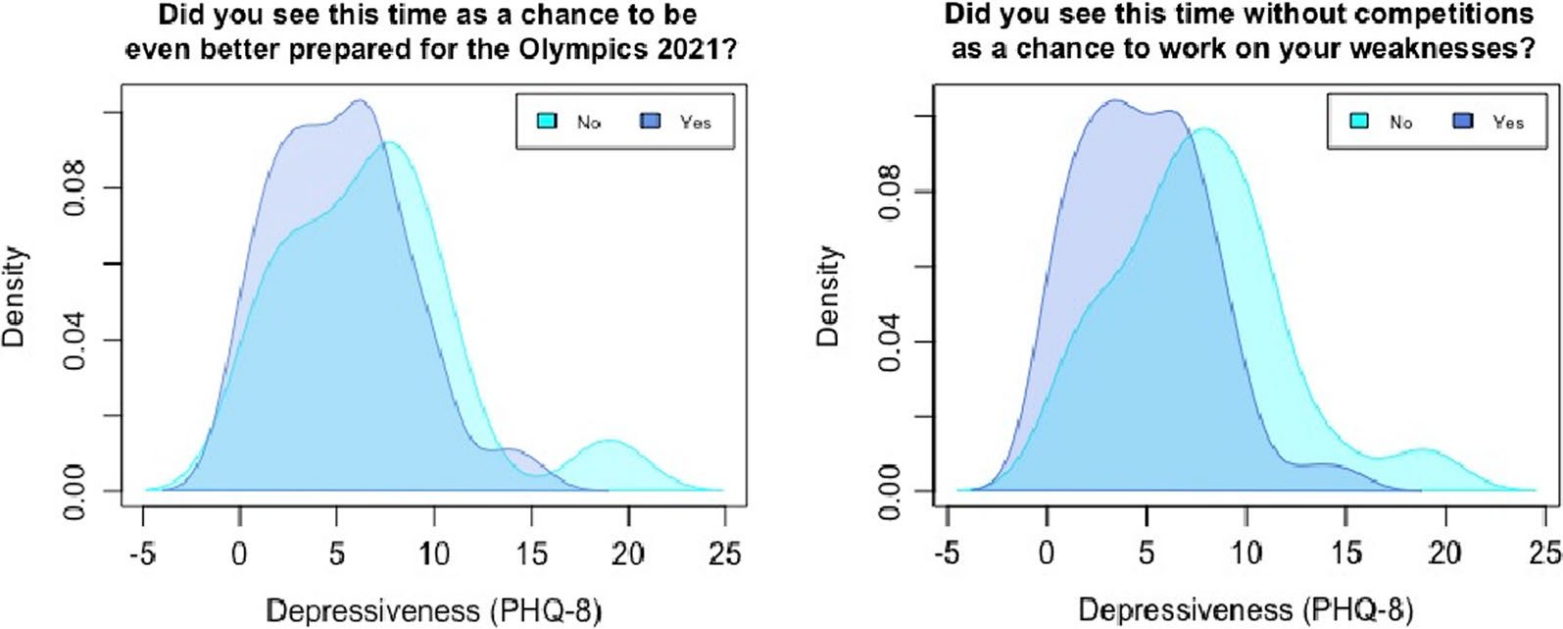
Density distribution—positive coping reaction by athletes to see the cancelation of the Olympic games as a chance

Regarding density distributions of the single items addressing the chances athletes can draw from the COVID-19 pandemic and the Tokyo 2020 Olympics postponement (Fig. 4), the density distribution of people answering “yes” (dummy coded variables) shifted to the left, suggesting lower scores on the PHQ-8. In Table 3 the mean difference of athletes in their depressiveness is demonstrated.

**Table 3:** Mean differences of athletes in their depressiveness (dummy coded variables)

	“Yes”		“Rarely”				
	*M*	*SD*	*M*	*SD*	*t*(82–83)	*p*	*g*
1. “Was it difficult for you when the Tokyo Olympics 2020 were postponed?”	6.36	3.79	3.93	2.96	− 3.04	< 0.003	− 0.68
2. “Did the COVID-19 crisis affect your training?”	5.56	3.76	4.60	2.41	− 0.56	= 0.58	− 0.26
3. “Did you find training more stressful?”	6.71	3.88	4.71	3.37	− 2.52	< 0.01	− 0.55
4. “Have you been afraid that athletes from other countries could train better during this time?”	6.65	3.84	3.94	2.86	− 3.57	< 0.001	− 0.78
5. “Have you been afraid that due to your different training you will not be at the same level like your opponents?”	7.06	3.66	3.49	2.63	− 5.03	< 0.001	− 1.09
6. “Did you feel pressure from yourself to get back to “Full-Contact-Training?”	6.34	3.49	3.90	3.59	− 3.03	< 0.003	− 0.69
7. “Did you feel less motivated?”	6.35	3.61	4.36	3.53	− 2.53	< 0.01	− 0.55
8. “Did you feel irritable, angry or aggressive?”	8.03	3.89	4.06	2.68	− 5.57	< 0.001	− 1.24
9. “Did you worry a lot about your performance after COVID-19?”	6.78	3.73	3.69	2.79	− 4.15	< 0.001	− 0.91
10. “Are you afraid of the first competition after this break?”	6.72	3.86	4.52	3.30	− 2.81	< 0.01	− 0.61
11. “Have you been afraid that you could lose your income due to the COVID-19 Crisis?”	6.76	4.49	4.54	2.60	− 2.86	< 0.005	− 0.62
12. “Have you been worried about life after your sports career?”	6.50	3.74	4.70	3.49	− 2.29	< 0.03	− 0.50
13. “Did you see this time as a chance to be even better prepared for the Olympics 2021?”	5.23	3.43	6.80	4.65	1.51	= 0.14	0.43
14. “Did you see this time without competitions as a chance to work on your weaknesses?”	4.85	3.24	7.65	4.31	3.12	< .002	0.71

Note *p < 0.05, **p < 0.01

## Discussion

The aim of this paper was to explore and describe mental health symptoms and emotional responses of Olympic athletes in light of the COVID-19 pandemic. It was hypothesized that the postponement of the 2020 Tokyo Olympics would have a negative impact on athletes and their well-being. This exploratory assumption was confirmed in several ways, although we acknowledge that the results need to be interpreted in the context of several limitations, including the relatively small sample size, the low statistical power, and the use of modified outcome measures.

Our results demonstrate a significant association between self-reported depression severity and athlete reactions to the postponement of the Tokyo Olympics, including perceived difficulties with training, motivation and self-regulation. These findings are consistent with other COVID-19 research regarding loss of motivation, coping problems and negative emotional response in elite athletes [5]. Our descriptive findings can be also interpreted as being consistent with the statement of the International Society of Sport Psychology, that the postponement of the games can lead to a loss of athlete motivation, meaning and identity [1]. Studies described signs of depression in elite athletes after an injury or after retirement [15, 16]. According to Schwenk et al. an explanation for the depressive symptoms could be the loss of devotion for the athletic competition and its resulting reward [16]. With the sudden cancelation of the games due to the COVID pandemic the training structure, the devotion in training and the dream of Olympic glory are taken away all at ones. This situation can be overwhelming for the athlete and could be a reason for the increased incidence of depressive symptoms in our study.

Our results suggest that athletes reported experiencing a range of problems associated with the postponement of the Tokyo 2020 Olympics. Athletes with higher depression scores reported experienced more impairing problems with the cancellation of the Games, including stress in training, and other motivational and self-regulatory aspects. Moreover, these athletes viewed the cancellation of the Olympic Games less as a chance to improve their performance compared to those screening below the PHQ-8 cut-off.

Very few Olympic athletes reported receiving professional support for their mental health and well-being at the time of responding. This is particularly surprising, since Olympic athletes are the highest performing athletes, who should be able to access and receive the best interdisciplinary support possible [10]. It is unclear whether or not athletes were offered mental health support by their sport, but examples suggest that the mental health needs of athletes are poorly recognized and responded to sporting federations, with a tendency only to promote *physical* health and advise amongst medical services [17], despite evidence that athlete mental health is impaired (e.g. in surfers [18], yet the International Surfing Association’s medical commission fails to refer to mental health care). The neglect of mental health and well-being, compared to the physical health needs of athletes must be addressed by sporting federations and associations. In 2019 the International Olympic Committee released a consensus statement to address mental health symptoms and disorders in elite athletes [11]. While mental health concerns and athlete well-being is discussed and considered important in agendas for Olympic societies, stakeholders and federations, there is a need for practical implementation and accessibility of psychological support for athletes [10, 11]. Highlighting the importance of mental health but inadequately supporting mental health intervention does little to help athletes nor federations [19].

The impact of the pandemic on elite athletes exposes the weak spots of systems and organizations in relation to mental health support. The whole multidisciplinary entourage (Sport-Physicians, Psychiatrists, Psychologists, Physiotherapists, Coaches and Mental Performance Consultants) in elite sports should be aware and prepared for mental health issues of their athletes, and national sports federations and stakeholders should create sports environments that promote mental well-being for the athletes and entourage members [11].

## Limitations

In addition to the limitations we already highlighted, this study has several other limitations. Unfortunately, we did not include para-athletes or athletes from developing nations making this sample less representative. Further limitations relate to the cross-sectional study design and the reliance on self-report. Nonetheless, we see value in research on real aspects of behavior as seen in the Olympic sports represented in this study, and in highly selective samples of Olympic athletes (24 different countries and 11 different sports) like ours. It can provide clues to how athletes react to and cope with live changing events and setbacks, not only in the lab but also in real life.

## Conclusion

The study demonstrated that the present sample of Olympic athletes suffered from the uncertainty associated with the postponement of the Olympic games. Only a small amount of athletes were receiving professional help regarding mental health issues in this difficult times. Sports federations should support the athletes and offer them, for example, sports psychological support in order to be able to better deal with the uncertainties and changes.

## Data Availability

The datasets used and/or analysed during the current study are available from the corresponding author on reasonable request.
